# Stress Radiographs in the Posterior Drawer Position at 90° Flexion Should Be Used for the Evaluation of the PCL in CR TKA with Flexion Instability

**DOI:** 10.3390/jcm11041013

**Published:** 2022-02-15

**Authors:** Lukas B. Moser, Matthias Koch, Silvan Hess, Ponnaian Prabhakar, Helmut Rasch, Felix Amsler, Michael T. Hirschmann

**Affiliations:** 1 Department of Orthopaedic Surgery and Traumatology, Kantonsspital Baselland (Bruderholz, Liestal, Laufen), Bruderholz, CH-4101 Basel, Switzerland; matthias.koch@ksbl.ch (M.K.); michael.hirschmann@unibas.ch (M.T.H.); 2 Research Group Michael T. Hirschmann, Regenerative Medicine & Biomechanics, Department of Clinical Research, University of Basel, CH-4001 Basel, Switzerland; silvanhess@msn.com; 3 Department of Trauma Surgery, University Medical Centre Regensburg, 93053 Regensburg, Germany; 4 Department of Orthopaedics, Trauma and Arthroplasty, Care Superspeciality Hospitals, Nampally, Hyderabad 500 001, India; drprabhakar1@gmail.com; 5 Institute of Radiology and Nuclear Medicine, Kantonsspital Baselland (Bruderholz, Liestal, Laufen), Bruderholz, CH-4101 Liestal, Switzerland; helmut.rasch@ksbl.ch; 6 Amsler Consulting, CH-4059 Basel, Switzerland; felix.amsler@amslerconsulting.ch

**Keywords:** cruciate retaining, CR, total knee arthroplasty, TKA, posterior cruciate ligament, PCL, stress radiographs, flexion instability

## Abstract

The purpose of this study was to define a cut-off value for the posterior drawer position in stress radiography that confirms an insufficiency of the posterior cruciate ligament (PCL) in cruciate-retaining (CR) total knee arthroplasty (TKA). In this retrospective study, 20 symptomatic patients with flexion instability and suspected PCL insufficiency in CR TKA were included. Asymptomatic patients served as an age- and sex-matched control group. All of the patients had undergone stress radiography, and the posterior translation was measured in a posterior drawer position at 30° and 90° flexion. The two groups were compared using t-tests and chi-square tests. The stress radiographs showed significantly more posterior translation in the symptomatic group (*p* < 0.01). Stress radiographs at 90° flexion more effectively discriminated between the patients with and without PCL insufficiency compared with those carried out at 30° flexion. Sensitivity and specificity testing revealed the best sensitivity (90.5%) and the best specificity (94.7%) at 90° posterior drawer radiographs at a cut-off value of 10 mm. Stress radiographs including the posterior drawer position at 90° flexion should be part of the diagnostic algorithm in patients with suspected flexion instability. A posterior translation of more than 10 mm in CR TKA strongly indicates an insufficiency of the PCL.

## 1. Introduction

Tibiofemoral instability after primary total knee arthroplasty (TKA) is responsible for 10–22% of TKA revision surgeries [1,2,3]. The most common causes for instability are the malposition of the implant, the fracture of the polyethylene insert, excessive soft-tissue release, a flexion–extension gap mismatch, or an insufficiency of the posterior cruciate ligament (PCL) in cruciate-retaining (CR) TKA [4,5]. 

In CR TKA, the PCL limits posterior tibial translation (PTT) during flexion and extension [3,6]. Hence, the integrity of the PCL is essential, and the ligament must not be sacrificed [7]. CR implants should be avoided when PCL insufficiency is present, and in patients with an increased risk of developing a posterior instability. In these cases, a posterior stabilized (PS) implant which substitutes the PCL should be utilized [8]. However, even when the PCL is intact before surgery, some conditions can reduce the integrity of the PCL during or after surgery. Direct iatrogenic damage can occur during primary TKA by harming or even resecting the PCL. Some studies have shown that the insertion of the PCL can be damaged when performing the tibial cut [9,10,11]. Indirect iatrogenic damage can occur if the PCL and the collateral ligaments are not well balanced. Despite this, a careful review of the current literature reveals that reports on PCL insufficiency in patients after primary CR TKA are rare. This might be because clinical examination and standard radiographs are often not sensitive enough to detect a PCL insufficiency [4]. Stress radiographs (30° and 90° flexion) are the gold standard in the quantification of posterior instability [12,13,14]. As the PCL performs its stabilizing function mainly between 60° and 120° flexion, posterior drawer testing at 30° flexion may not accurately show anteroposterior instability [15]. Stress radiographs at 90° flexion could help to identify the instability and quantify the amount of posterior translation. 

At present, however, no cut-off value for posterior translation in patients with flexion instability has been defined. Thus, the current study aims to compare the posterior translation of symptomatic patients suffering from flexion instability and suspected PCL insufficiency with an asymptomatic control group using stress radiographs. It was hypothesized that a posterior drawer position at 90° flexion would better reveal the instability than one at 30° flexion. A cut-off value confirming PCL insufficiency in CR TKA knees will be presented based on the findings of the present study.

## 2. Materials and Methods

The hospital registry was retrospectively searched for patients with CR TKA suffering from symptomatic posterior instability and suspected PCL insufficiency (symptomatic group). A total of 20 patients (male:female, 8:12; right:left, 10:10; mean age ± standard deviation (SD) 63 ± 5 years; range 53–73 years) met the inclusion criteria.

Additionally, the registry database was searched for patients with CR TKA without symptomatic flexion instability to serve as an age- and sex-matched control group (the asymptomatic group) (Table 1). Ultimately, 20 patients (male:female, 8:12; right:left, 12:8; mean age 63 ± 8 years; range 50–76 years) were included. 

All of the symptomatic and asymptomatic patients had undergone stress radiographs in the posterior drawer position at 30° and 90° flexion. The mean time from the primary TKA to stress radiographs was 44 ± 43 months in the symptomatic group and 31 ± 26 months in the control group. There were no significant differences in patient age at the time of the surgery, or in the timespan between the surgery and stress radiographs between both groups (Table 1).

**Figure 1:**
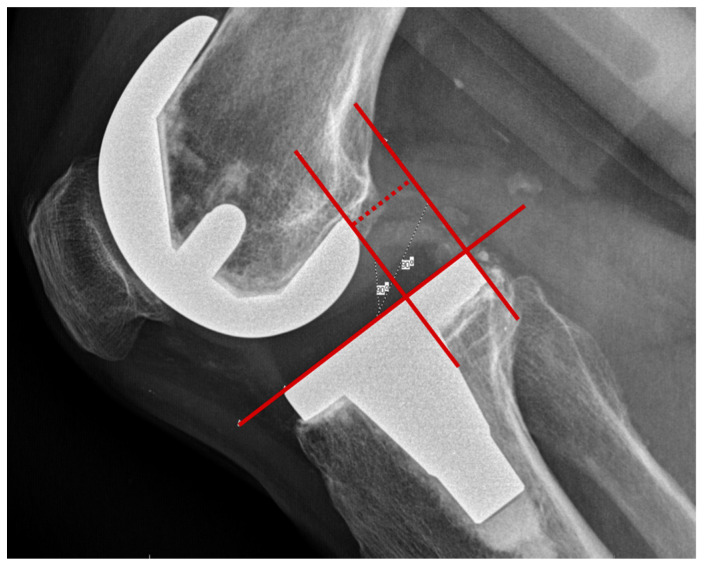
Measurement technique of the posterior tibial translation in stress radiographs in approximately the 90° posterior drawer position.

The stress radiographs were taken in a true lateral view in the posterior drawer position at 30° and 90° flexion with an applied posterior force of 15 kPa using the Telos device (Telos GmbH, Marburg, Germany). The posterior translation of the tibia was evaluated by measuring the translation of the posterior tibial component in relation to the posterior component of the femur condyle (Figure 1). All of the measurements were performed with the PACS iSite Web Version 4.1.144.0 (Picture Archiving Communication System, Phillips Easy Vision, Amsterdam, The Netherlands). A tangent line was drawn along the plateau of the tibial component. Perpendicular to the tibial component, two lines were drawn passing the posterior part of the tibial component and the most posterior part of the femoral component. The distance between these two lines was measured in millimetres, representing the posterior translation of the tibia. 

In order to observe intra- and inter-rater reliability, two independent observers evaluated all of the stress radiographs twice, with a two-week interval, in random order. One rater was an orthopaedic surgeon with several years of clinical experience in the evaluation of posterior stress radiographs, and the other was considered a novice rater. The measurement technique was explained to the novice rater prior to the conducting of the study. 

All of the patients gave their informed consent, and the study was approved by the local ethical committee (EKNZ, 2018-01371). All of the procedures performed were in accordance with the ethical standards of the institutional and/or the national research committee, and with the 1964 Declaration of Helsinki and its later amendments or comparable ethical standards. The data were analyzed using IBM SPSS Statistics for Windows, version 26.0 (IBM Corp: Armonk, NY, USA). Intra- and inter-rater reliability were tested with the intra-class correlation (ICC) for both groups separately and overall; single and average measure values with 95% confidence intervals (CI) are shown. According to Rosner et al., the reliability by means of ICC is classified as follows: >0.75 = excellent, 0.75 to 0.44 = fair to good, and <0.4 = poor [16]. Continuous data are described as the mean and SD, and the categorical data are described as frequencies and proportions. 

The single-measure ICC for the intra-rater reliability of the total sample were between 0.95 and 0.99, and the average-measure ICC was between 0.97 and 1.00, which can be interpreted as excellent (Table 2). The inter-rater reliability was between 0.79 and 0.91 for the single-measure ICC, and between 0.88 and 0.95 for the average-measure ICC; therefore, they can be interpreted as good to excellent (Table 3). The reliabilities of the subsamples were only marginally lower compared to the total sample.

The two groups were compared with t-tests (continuous variables) and chi-square tests (nominal variables). The *p*-values were two-sided, and were considered statistically significant if they were smaller than 0.05. The specificity and sensitivity were calculated according to the following formulae: specificity = the number of true negatives/(the number of true negatives + the number of false positives), and sensitivity = the number of true positives/(the number of true positives + the number of false negatives).

## 3. Results

The stress radiographs at 30° and 90° flexion showed significantly more posterior translation in the symptomatic group than in the asymptomatic group (*p* < 0.01) (Table 4). The mean posterior translation was 6.5 ± 4.2 mm(30° flexion) and 15.2 ± 3.3 mm (90° flexion) for the symptomatic group, and 2.6 ± 3.5 mm (30° flexion) and 6.5 ± 2.5 mm (90° flexion) for the control group.

**Figure 2:**
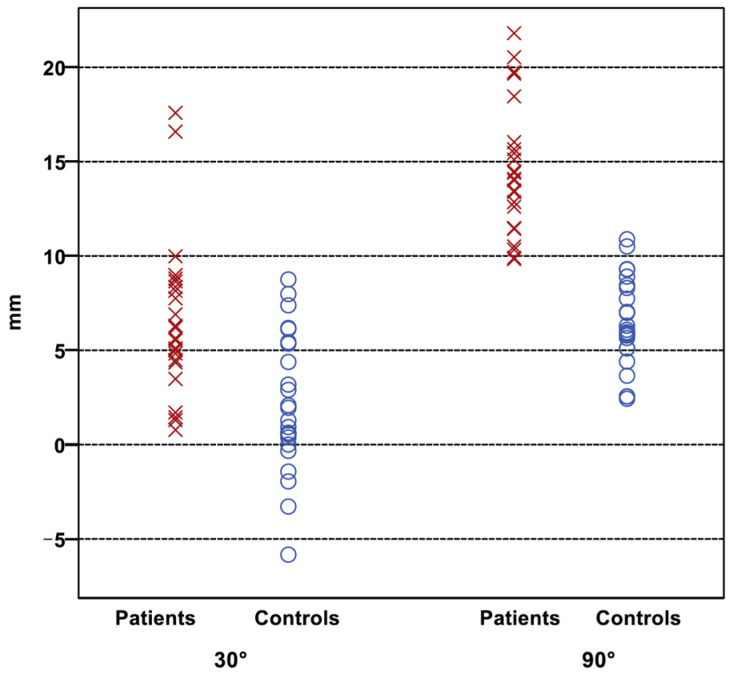
Distribution of the PTT of the patients (cross) and the control group (circle). There is a better discrimination between both groups at 90°, indicating a cut-off value at 10 mm.

Stress radiographs at 90° flexion better discriminate between knees with PCL insufficiency and knees without PCL insufficiency than those at 30° flexion (Figure 2).

Sensitivity and specificity testing revealed the best sensitivity (90.5%) and the best specificity (94.7%) at 90° posterior drawer radiographs at a cut-off value of 10 mm (Table 5).

## 4. Discussion

The most important findings of the present study were the following:

Firstly, the high intra- and inter-rater reliability indicate that the posterior translation of the tibial prosthetic component can be measured reliably on stress radiographs in the posterior drawer position. Even the single-measure ICC is so high that an inexperienced clinician can reliably measure the clinical evaluation of posterior translation. This finding is in line with previous studies using stress radiographs for the evaluation of the integrity of the PCL in native knees [17,18]. 

Secondly, patients with symptomatic flexion instability had an increased posterior translation compared to the control group. The difference between the groups was significant at both angles of flexion (*p* < 0.001). However, measuring the posterior translation at approximately 90° flexion enables a better discrimination between the symptomatic patients and the control group. This can be explained by the fact that the PCL performs its stabilizing function mainly between 60° and 120° flexion, and only ongoing flexion reveals the effect of PCL insufficiency on anterior–posterior instability.

Some studies compared the anterior–posterior laxity of CR and PS TKA at 20–30° and 60–90° flexion angles [17,18,19,20]. However, most of the studies relied on manual devices such as the rolimeter or the arthrometer for the evaluation of the overall anterior–posterior translation, and did not independently investigate the posterior translation during the clinical examination [19,20]. However, as these measurements are highly operator-dependent, stress radiographs have proven to be the most accurate measurement technique for the evaluation of a posterior tibial translation [21]. 

Dejour et al. investigated the laxity of 118 PS and 138 cruciate-sparing prostheses in stress radiographs [17]. They compared the clinical results with the measurements of laxity at 70° flexion at a mean follow-up of 4 years. Only six patients (5%) of the PS group had an anterior–posterior laxity of 5–10 mm. In contrast, 57 patients (41%) of the cruciate-sparing group had a translation between 5 and 10 mm, and three patients (2%) had a laxity of more than 10 mm. Measurements on the stress radiographs showed significantly more laxity in the anterior (7 ± 5 mm vs. 3 ± 3 mm in 70° flexion) and posterior direction (8 ± 4 mm vs. 4 ± 2 mm in 70° flexion) of the cruciate-sparing groups. The mean posterior translation is comparable with the mean measurements of the control group of the present study (6.5 ± 2.5 mm). The three patients with a translation of more than 10 mm could have suffered from PCL insufficiency. However, the authors did not investigate this.

Seon et al. investigated the effect of anterior–posterior laxity on the knee function and range of motion in 55 patients following CR TKA [18]. They performed their measurements using anterior and posterior stress radiographs at 90° flexion. Thirty-eight patients had an anterior–posterior translation of less than 10 mm (mean 7.6 mm) and were regarded as stable, whilst 17 patients with more than 10 mm laxity (mean 13.1 mm) were identified as unstable. The stable patients had a better functional outcome and increased flexion. Although the findings of the unstable group appear similar to the present study, the limit of 10 mm for the definition of the stability of the knee was chosen arbitrarily in this study. Furthermore, they did not distinguish between anterior and posterior translation.

Other authors measured the posterior translation manually using an arthrometer and/or rolimeter. Jones et al. studied the effect of sagittal laxity on function after CR TKA with two different tibial inlays (flat and anteroposterior lipped) [19]. A total of 97 knees were included and assessed at 30° and 75° flexion using a KT 1000 arthrometer. Laxity was 7.3 ± 4.0 mm at 30° and 4.6 ± 3.1 mm at 75° flexion. The posterior translation was 1.5 ± 0.9 mm at 30° and 1.7 ± 1.7 mm in 75° flexion. Interestingly, this contradicts the present study, which found increased posterior translation in increased flexion. One reason for the different findings might be difficulties in detecting posterior translation associated with the use of the arthrometer. Furthermore, Jones et al. generated three groups based on total laxity (0–5 mm, 5–10 mm, and more than 10 mm). Patients with a laxity of more than 10 mm showed significantly less flexion and lower KSS scores than the other groups. The authors concluded that a sagittal laxity of 5–10 mm should be regarded as optimal. However, the cut-off value for the groups was chosen arbitrarily. 

Matsuda et al. evaluated the sagittal knee stability of 19 knees (CR TKA) using a KT-2000 arthrometer [20]. At an average of 9 years follow up, the mean anterior–posterior translation was 10.1 ± 4.2 mm at 30° and 8.0 ± 3.1 mm at 75° flexion. Seven knees showed an increase of translation at 75° flexion compared to 30° flexion, and were defined as PCL insufficient knees. The present study does not support these findings, as both symptomatic and asymptomatic patients with CR TKA had increased posterior translation at 90° flexion compared to 30° flexion. A possible explanation for this mismatch might be that Matsuda et al. evaluated the anterior–posterior translation and not the isolated posterior translation.

However, none of these studies compared their findings on symptomatic patients with a matched asymptomatic control group of the same prosthetic design. The present study fills that gap. A cut-off at 10 mm showed the best sensitivity (90.5%) and specifity (94.7%). Based on this, clinicians will be able to assess the integrity of the PCL in a CR TKA using stress radiographs without relying on arbitrary benchmarks. A posterior translation of more than 10 mm at 90° flexion in the posterior drawer position indicates an insufficient PCL in a CR TKA.

This study has a considerable number of limitations. Firstly, CR prostheses from different manufacturers were included in this study. Differences in design might have influenced the posterior translation. The analysis of the subgroups was not possible due to the small sample size. Nevertheless, the statistically significant findings of the present study suggest that this aspect does not play a key role in the particular study question.

Secondly, the control group of patients was not completely asymptomatic, and included patients with painful knees but without flexion instability. However, only patients without reported or suspected posterior instability were included. Therefore, no impact on posterior translation in stress radiographs was expected.

Thirdly, there was a time difference in the mean time from the primary TKA to the stress radiographs between the symptomatic and the control group (44 ± 43 months: 31 ± 26 months). Although we can assure that the patients of the control group were not treated for flexion instability at our clinic after the same follow-up as the symptomatic group, those patients could still have developed flexion instability and been treated at a different clinic.

Fourthly, the posterior translation might be difficult to interpret in cases when the knee was slightly twisted and the radiograph was not shot perfectly laterally. However, the good inter- and intra-rater testing has proven that the stress radiographs are a reliable method for the evaluation of the posterior translation. Stress radiographs only investigate the stability of the knee under static conditions. It would have been interesting to investigate the stability in a dynamic setting using fluoroscopy, which was not performed in the present study. 

## 5. Conclusions

Stress radiographs including the posterior drawer position at 90° flexion should be included in the diagnostic algorithm in patients with flexion instability. A posterior translation of more than 10 mm in CR TKA strongly indicates an insufficiency of the PCL.

## Figures and Tables

**Table 1:** This table illustrates the patient demographics of the symptomatic and the matched control group.

	Symptomatic Group	Control Group	*p*-Value
Male	12 (60%)	12 (60%)	1.000
Female	8 (40%)	8 (40%)	1.000
Patient age: Primary TKA Mean ± SD	58.9 ± 6.9	60.7 ± 8	0.824
Patient age: Stress-X-ray Mean ± SD	62.6 ± 5.4	63.3 ± 7.6	0.738

**Table 2:** Intra-rater reliability of the measurements of posterior translation for the patients, the control group, and all of the patients together.

Variable	Group	Description				ICC		95% CI	
		Measure	N	Mean	SD	Method	Value	Lower	Upper
30° Rater 1	Total	1	40	4.09	4.72	single	0.99	0.97	0.99
		2	40	4.18	4.84	average	0.99	0.99	1.00
	Patients	1	20	6.17	4.31	single	0.97	0.93	0.99
		2	20	6.25	4.48	average	0.99	0.97	1.00
	Controls	1	20	2.13	4.35	single	0.99	0.97	1.00
		2	20	2.22	4.45	average	0.99	0.99	1.00
30° Rater 2	Total	1	40	4.98	4.44	single	0.95	0.90	0.97
		2	40	4.90	4.43	average	0.97	0.95	0.99
	Patients	1	20	6.80	4.47	single	0.93	0.84	0.97
		2	20	6.77	4.53	average	0.97	0.91	0.99
	Controls	1	20	3.16	3.68	single	0.94	0.84	0.97
		2	20	3.02	3.51	average	0.97	0.92	0.99
90° Rater 1	Total	1	40	10.58	5.44	single	0.99	0.98	1.00
		2	40	10.60	5.44	average	1.00	0.99	1.00
	Patients	1	20	14.99	3.39	single	0.98	0.94	0.99
		2	20	14.84	3.57	average	0.99	0.97	1.00
	Controls	1	20	6.18	2.91	single	0.98	0.95	0.99
		2	20	6.35	3.17	average	0.99	0.97	1.00
90° Rater 2	Total	1	40	10.98	5.25	single	0.98	0.95	0.99
		2	40	11.12	5.57	average	0.99	0.98	0.99
	Patients	1	20	15.15	3.96	single	0.95	0.89	0.98
		2	20	15.72	3.63	average	0.98	0.94	0.99
	Controls	1	20	6.81	2.10	single	0.88	0.72	0.95
		2	20	6.52	2.45	average	0.94	0.84	0.97

**Table 3:** Inter-rater reliability of the measurements of posterior translation for the patients, the control group, and all of the patients together.

Variable	Group	Description				ICC		95% CI	
		Rater	N	Mean	SD	Method	Value	Lower	Upper
30°	Total	1	40	4.13	4.76	single	0.79	0.64	0.88
		2	40	4.94	4.37	average	0.88	0.78	0.94
	Patients	1	20	6.21	4.37	single	0.86	0.69	0.94
		2	20	6.79	4.42	average	0.93	0.81	0.97
	Controls	1	20	2.06	4.30	single	0.59	0.21	0.82
		2	20	3.09	3.54	average	0.74	0.35	0.90
90°	Total	1	40	10.59	5.43	single	0.91	0.83	0.95
		2	40	11.05	5.38	average	0.95	0.91	0.97
	Patients	1	20	14.91	3.46	single	0.71	0.40	0.87
		2	20	15.43	3.75	average	0.83	0.57	0.93
	Controls	1	20	6.26	3.03	single	0.76	0.48	0.90
		2	20	6.67	2.21	average	0.86	0.65	0.95

**Table 4:** This table shows the measured posterior translation using stress radiographs at 30° and 90° flexion.

	Symptomatic Group	Control Group	*p*-Value
	Mean ± SD (in mm)	Mean ± SD (in mm)	*p*
30° Flexion	6.5 ± 4.2	2.6 ± 3.5	0.000
90° Flexion	15.2 ± 3.3	6.5 ± 2.5	0.000

**Table 5:** Sensitivity and specificity for the detection of PCL insufficiency at a cut-off value of 10 mm at 90° flexion.

	Affected	
	No	Yes
<10 mm	18 (90%)	1 (5%)
≥10 mm	2 (10%)	19 (95%)
Total	20 (100%)	20 (100%)

Specificity: 94.7%; sensitivity: 90.5%.

## Data Availability

The data is not publicly available in accordance with the approval of the local ethical committee.
